# Experiences of Disabled Older Adults in Tokyo’s Adult Day Care Centers during COVID-19—A Case Study

**DOI:** 10.3390/ijerph19095356

**Published:** 2022-04-28

**Authors:** Takashi Naruse, Masakazu Hatsushi, Junichi Kato

**Affiliations:** 1 Division of Care Innovation, Global Nursing Research Center, Graduate School of Medicine, The University of Tokyo, Tokyo 113-0033, Japan; 2 Hakononakanohako, General Incorporated Association, Tokyo 140-0001, Japan; ru20150714@gmail.com; 3 Long-Term Care Health Facility, Peace Plaza, Seishinkai Medical Corporation, Tokyo 183-0021, Japan; junichi050@gmail.com

**Keywords:** adult day care, aged care, long term care, client experience, COVID-19

## Abstract

The COVID-19 pandemic resulted in social isolation among elderly people with disabilities. Adult daycare (ADC) is an important community care option for socialization among people with disabilities. However, their experiences with ADC remain underexplored. Thus, this study investigated the experiences of community-dwelling disabled elderly with ADC from the perspective of socialization. Four older women from Tokyo with disabilities, availing of one ADC service, were interviewed across two sessions between November 2020 and January 2021. The transcribed interviews and field notes were analyzed qualitatively. This yielded eight categories: two pertaining to context (“restricted social interaction outside of ADC”, “feeling simultaneously grateful and ashamed of oneself as a recipient of care services”), and six pertaining to experience with ADC (“take a catastrophic defensive posture in situations where one’s perception of value is shaken”, “express oneself positively to justify one’s daily life”, “have trouble knowing what to do”, “put oneself in a shaded exchange relationship”, “examine the value of elderly people in need of care in society”, and “savor regular contact with others”). Ensuring the use of ADC as a safe place for interaction while considering pandemic-related needs is important to develop policy and practical responses to restricted socialization during COVID-19.

## 1. Introduction

Following the spread of COVID-19, the daily activities of diverse people, especially those with disabilities, were restricted. This limited access to long-term care services. Adult daycare (ADC) is a generic term for eldercare; it comprises building-based services that offer various programs and amenities to older people [1]. In Japan, ADC is a type of long-term care (LTC) service. In April 2018, with 1.13 million ADC clients, ADC was the most popular type of LTC service among the Japanese disabled population [2]. Intervention-related benefits include improved physical, mental, and social functioning, exposure to comprehensive care, improved well-being, and alleviation of family caregivers’ burdens [1,3,4]. Gaugler [5] warned about the possibility that COIVD-19 might inhibit the use of ADC by older people. He suggested that this was because of the absence of clear evidence of service effects from a randomized control design. Prior literature has revealed that it is difficult to measure service use benefits among ADC clients because of the heterogeneity in their needs [6]. Gaugler also mentioned that numerous older adults hope to live in their homes and need ADC, arguing for the necessity of enhancing and confirming the significance of ADC in the society [5].

Japanese clients’ experiences during adult daycare service use (J-AdaCa Tool) was developed to assess the richness of client experiences with ADC service use [7]. It was developed based on the Japanese ADC logic model that ADC staff and researchers had developed through repeated interviews and discussions [8], and the tool requires staff evaluation of each client. It comprises four factors regarding the experience of ADC use: “social participation”, “hygiene and health”, “exercise and eating habits”, and “family support.” This is in line with an international review that reported four general aims of ADC, namely providing social and preventive services, supporting clients’ continued independence, supporting clients’ health and daily living needs, and enabling family caregivers to take breaks from daily care or continue with their employment [1]. Using the J-AdaCa Tool [7], we implemented a cross-sectional survey that asked ADC staff about their clients’ service needs [9]. Among the four kinds of ADC-related experience, “social participation” was the most common objective for 75% of ADC clients, and it might be important to fulfill the clients’ socialization needs [9]. 

Fulfilling socialization needs can lead to improved health because social participation improves clients’ physical and mental health and promotes positive social relationships [10]. Owing to the ongoing pandemic, older adults with disabilities are expected to limit their social activities out of ADC, and ADC would have a more significant value on their quality of daily life. However, limited reports have shown clients’ views on experiences with ADC during COVID-19 [11]. This case study aimed to describe the experiences of clients with ADC, focusing on their social participation. The description of clients’ subjective experiences would indicate that ensuring the use of ADC as a safe place for interaction while considering the new needs that emerge due to the pandemic is vital for the development of policy and practical responses to restricted socialization during COVID-19. Thus, this study was expected to provide an insight into the current situation of ADC clients, contributing to subsequent case accumulation and deeper analysis.

## 2. Materials and Methods

### 2.1. Study Design

This qualitative study was based on Yin’s case study method [12]. In the case study method, questions, such as “why” and “how”, are presented; these are useful when focusing on current phenomena, and multiple cases can be analyzed. Yin defines the case study research method as an inquiry that deals with a technically distinctive situation [12]. In such a situation, there will be many more variables of interest than data points that rely on multiple sources of evidence, with data needing to converge in a triangulating fashion. Further, the case study method benefits from the prior development of theoretical propositions that guide data collection and analysis. Multiple case analyses both within each case and across cases can be used to either augur contrasting results for expected reasons or augur similar results in the studies. Multiple case studies in a single site were adopted because such an approach is suitable for explaining ADC experience in the context of COVID-19. “Case” was defined as an individual person in this study.

### 2.2. Participants

The target facility (ADC-X (one adult daycare facility and its name was blinded and written as X)) was selected based on the fact that it was an average-sized facility in Japan and that there was no bias in the disabilities or diseases of the users. The researcher contacted the administrator directly, explained the purpose and content of the study in writing, and obtained their consent. In response to the researcher’s request, the administrator selected candidate cases for interviews and arranged the date, time, and location for their introduction to the researcher. The researcher, who received the introduction from the manager, explained the purpose and content of the study in writing directly to the candidates. An interview survey was conducted only after consent was obtained.

The interviewees were elderly users of ADC-X who were able to conduct individual interviews with the researcher in Japanese. In selecting the candidates, we asked them to be introduced to those who were most likely to be willing to meet the researcher in person during the COVID-19 pandemic.

Consent was confirmed in writing. The office that participated in the interview survey was given 5000 yen, and the participants were given 1000 yen worth of library cards. This study was approved by the Ethics Committee of the Graduate School of Medicine and Faculty of Medicine at the University of Tokyo (Issue No. 2019037NI).

### 2.3. Participant Facility (ADC-X)

**Figure 1:**
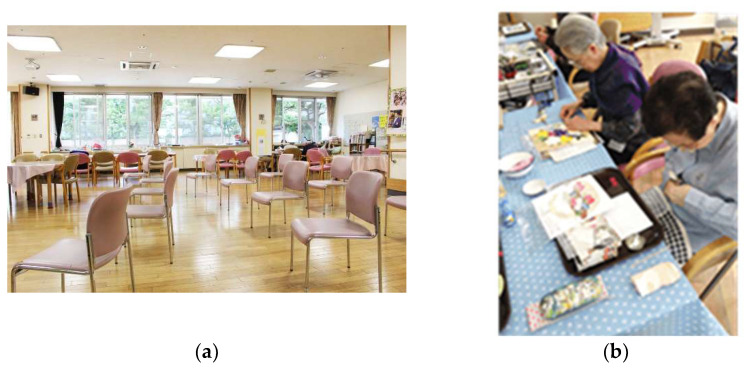
Photograph of ADC-X: (**a**) living and exercise space; (**b**) craft activity.

ADC-X is located in the central area of Tokyo. It is run by a social welfare corporation, has a capacity of 45 users, and is open from 08:45 to 17:30, Monday through Saturday. The facility has a total floor space of 529.00 m^2^ (including a dining room and functional training room of 198.6 m^2^). The facility has one administrator, two social counselors, ten long-term care staff, three nurses, two occupational/physical therapists, and one clerk. The average number of users per day is approximately 30. After COVID-19 spread, there were no clusters of infected patients in the facility, and the number of users has not changed significantly from before the pandemic (as of October 2020). Figure 1 shows the photos of ADC-X.

### 2.4. Data Collection

Before the interview, a face sheet was used to enquire about the participant’s age, use of ADC, and history of use. Subsequently, a semi-structured individual interview using an interview guide was conducted for approximately 60 min. The interviews were conducted twice, with an interval of at least one week. The reason for conducting repeated interviews was to speak at a slower speed so that the interviewee would feel comfortable and not get tired. This was also done so as to finish the interview within an hour. In addition, we considered that repeated interviews would help the participants get familiar with the researcher and the interview, making it easier for them to speak their mind.

In the interview guide, the interviewees were first asked (1) how they spent their day at the daycare center, (2) what they thought of the others (users and staff) at the center, (3) what kind of relationships they had with them, and what they thought about them. The data collection period was from October 2020 to January 2021. The interviews were recorded and transcripts in verbatim were used for subsequent analysis. The tone of voice and facial expressions of the interviewee during the recording were also referenced as data. The words obtained from the conversation at the end of the interview were also written with the interviewee’s consent.

In addition to the interviews, the first and second authors stayed on the floor of the target facility for four days (approximately two hours each) with the permission of the administrator. They observed the participants and people around them. In doing so, they were careful not to record any personal information other than of the study participants. What researchers noticed during the observation was recorded in the field note and used for the analysis. In observation, researchers asked ADC staffs about daily participants’ behavior and how staffs recognized them. All comments were added to field note as recorded data.

Two researchers were present during interviews. The first author conducted the interview, and the second author observed the interviewer during the interview as well as helped with ventilating, setting up panels for infection control, and monitoring participants’ physical condition.

### 2.5. Data Analysis

The first author conducted the analysis. First, a summary of the participants was compiled for each case. As basic information, age, activities of daily living (ADL), family members living with the patient, and history, frequency, and duration of ADL use were summarized. In addition, if there were any parts where thoughts regarding daily life were discussed, they were summarized as background information. Subsequently, for each case, we extracted what was said about others in the same ADC and the relationship between them and the participant. The content of what was said was extracted from raw data in a coherent manner, and a summary representing the content was created. Multiple summaries were classified by examining their similarities and differences, and codes were created to represent them. The created codes were integrated to represent meaning in the participant’s unique context while referring to their summary, and subcategories and categories were generated for each case. After the generation of categories was completed for all cases, the generated subcategories and categories were lined up for classification and integration to examine similarities and differences. An integrated version of the subcategories and categories was generated, ensuring no loss of meaning in each case. The first author’s analytical process was confirmed by the third author (an administrator of another ADC in Tokyo area in 2020 and experienced participants in the analytical process of qualitative research). The results agreed upon and integrated by both authors were reviewed by one administrator and two staff members of ADC-X as well as the second author, who was present at the interview to check for any discrepancies in wording. The results were supervised by an expert in qualitative research (a Japanese professor of gerontological nursing), and were finalized with the agreement of all authors. Originally, peer review was planned to be conducted on the participant herself, but due to the re-expansion of the COVID-19 epidemic, it could not be done for a year because researchers could not get in and out of the ADC. Furthermore, it was difficult for participants to communicate online because of her impaired vision and hearing. Therefore, a careful confirmation process was conducted by someone other than them, and the results were compiled.

Throughout the analysis process, we used the “Model of Social Interaction (SI model) [13]” for the development of the analytical framework. The model was developed to explain clients’ social interaction and help guide the assessment and intervention process of occupational therapists. Social enactment skills, the output of the process, are produced as a result of the individual’s ability to absorb and make sense of social information and develop a plan of action. This process is influenced by several variables, including the individual’s sensory organs, cognitive abilities, emotional state, volitional traits, and interactional style. All data collection and analysis referred to the model elements. The data that could be elements of the SI model were included in the analysis, and the others were excluded. In case of a doubt regarding whether a phenomenon met the definition of a model element, two researchers discussed and decided how to treat it. In order to describe clients’ experiences from their own perspectives, we did not break down the data into each element, but created categories based on their similarity.

## 3. Results

### 3.1. Participants

Participants were in their 80 s to 90 s, and all were female. The frequency of ADC visits was two or three times a week. Two participants, ID01 and ID02, had been using ADC for more than eight years, while the remaining two had been using the same for about one month. Three patients lived with their families, whereas only one lived alone. In order to avoid identification of who the case was by the staff of ADC-X, no further basic information is shown here. The participant’s ID were blinded and shown by ID01 to ID04 in this paper.

### 3.2. Qualitative Analysis

From the analysis of the interviews and field notes, eight categories merged, two as context and six as experience with ADC. 

#### 3.2.1. Context 

Table 1 shows the results of “Context.”

(1) Restricted Social Interaction Outside of ADC

All participants explained their situation using expressions such as “COVID-19 restrictions on going out” and “ADC is good place to stay.” However, other reasons also emerged. ID03 explained “dropping out of the old familiar” and “difficulty in communicating using information and communication technology (ICT)” because of aging.

“Gradually, we are left with fewer friends. Some of them pass away, and... (I sometimes receive a call saying that) they have been admitted to a long-term care institution. I had been able to meet them, talk to them, and have a normal conversation with them, but (now) I cannot see them or I am not allowed to see them anymore. I cannot get on the train, and they cannot either because they have been told not to go out. … (Communication without face-to-face communication) is like scratching your feet from the top of your shoes (laughs). It is frustrating; it is not like meeting someone at all.”

(ID: ID03)

ID02 showed “difficulties in interacting with others due to disability” and “difficulty in communicating with family and neighbors.”

“When your body is like ours, it is hard to stand still if you are standing there talking (outside of the house, on the streets). … If it is a family member, you can say what you want, you know. If it were my family, I would say what I wanted to say to my son, but if I said what I wanted to say to my daughter-in-law, I would be no good to her.”

(ID: ID02)

ID04 also expressed “difficulty in communicating with family and neighbors.”

“(Staying with other people in ADC) could be (a good place for me, because now) I am 84 years old. No one would pay attention to me, and my husband is long gone. If an old lady like me were to say welcome back to the children on their way home from elementary school, for example, she would get a strange look. ...I cannot even talk to them like that.”

(ID: ID04)

(2) Feeling Simultaneously Grateful and Ashamed Oneself as a Recipients of Care Services

All participants expressed their feelings through expressions such as being “grateful to live with access to care services” and “feeling patronized for living with access to care services.” ID01 and ID03 showed that they understood that their future is going to be much worse.

“(About ADC-X) I cannot think of anything better than just being taken care of with a ride home. ...I am grateful to the staff for their hard work in disinfecting and preventing infections so that I can visit without worry. I am grateful that I can come here without any concern. They let me live, they take care of me so much, and in the end, (the long-term care insurance system) are eating up the lives of young people in the future. It hurts my heart to even think about it.”

(ID: ID01)

#### 3.2.2. Experience within ADC

Table 2 shows the results of “Experience within ADC.”

(1) Take a Catastrophic Defensive Posture in Situations Where One’s Perception of Value Is Shaken

Participants talked about the painful experience of being confronted with the fact that they were disabled by receiving care at the ADC. Two participants talked about “Cynicism against the disabilities of others for self-protection.” ID01 and ID03 showed their cynical behavior toward other clients’ disabilities and problems. They argued their positions without problems, similar to others. They accepted their current disability by comparing it with other heavier clients.

“Someone comes here in the daytime and he/she sleeps... what can I say... (frowned)... sleeping, and (doing) nothing at all. ...The ADC staff often talk to them and tell them something. Also, I guess they have to clean up the toilet, so they talk to him/her and take him/her to the toilet. I think it is hard work for the staff, and I am just observing. ... I am not going to sleep. (I do not sleep. When I am at home, I tend to fall asleep, but here, I am wide (awake) … (At home), I tend to fall asleep, but I pull out my old books (and read them and try to stay awake). ”

(ID: ID01)

ID01 and ID04 were observed to be “producing self-compassion actively” within the ADC. She talked about her inferior feelings during the interview as well as with other ADC clients. In both observations, the researcher recognized that she spoke out her inferior feelings clearly in a loud voice. She behaved like a central person in the old women group (two to four persons). Two researchers recognized her as playing a tragic heroine and assessed her face as being comfortable.

“(About my physical disability), I feel inferior…and I want to die comfortably (with a quick wave of her hand up in the air) … I think so every day.”

(ID: ID01)

After each interview conversation, all four participants showed pleasure with the interview because they felt happy to freely express their negative feelings. Conscious of taking up too much staff time in daily ADC visits, they said, “This is a good time for saying negative recognition for myself. “Researchers observed that both participants (ID02 and ID03) did not express their negative feelings in ADC but did so in the interviews. “Producing self-compassion actively” was only observed in ID01 and ID04, however, other two participants looked like they were hoping to do that.

(2) Express Oneself Positively to Justify One’s Daily Life

ID03 talked about the willingness to be useful to others and explained the clients’ situation as useless. Three participants (ID01, ID02, and ID03) were observed helping other clients within the ADC. ID02 helped others prepare tools for the handicraft program, while ID03 facilitated the mahjong game that she was participating in and laid out chairs for other players. They smiled and were appreciated by others. These kinds of experiences were seen as being “happy to express and be recognized and that I am still useful and valuable” ID03.

“I want to tell them (staff and other clients) that I can do this much on my own!”

(ID: ID03)

Regarding the feeling of being happy to express and recognize that the self was still useful and valuable, ID01 explained that her staying in ADC could contribute to helping her daughters.

“When I am here, my daughter feels safe and will not have to come to my house. ...So, I think that coming here is also for my daughter’s sake, and for my sake, too.”

(ID: ID01)

ID02 and ID03 talked about their experience of “motivation to behave positively.” ID02 explained the motivation of other clients in ADC, while ID03 explained the motivation of the staff in ADC.

“The fact that there are places where young people are willing to work happily made me think that Japan is not yet abandoned. (Regarding the fact that the staff are trying their best to provide good service within the ADC despite the limitations of COVID-19) I feel like I have to work harder too.”

(ID: ID03)

ID02 explained, “Getting a seed to have a conversation with family members.”

“When I brought these things (her crafts) home and showed them to my daughter-in-law, she talked to me, and this became a conversation starter (smiling).”

(ID: ID02)

“Receiving care in the same place as other elderly people who need care, making it easier to accept one’s disabilities” was observed in all four participants. ID01 showed her comfort through peer conversations with other disabled people, and ID03 recognized that her disability was natural through receiving care and observing other people being taken care of.

“(When they call my name or congratulate me on my birthday), I feel that they have not forgotten me... I can feel the warmth of the staff. (With other clients), we have things in common, such as deafness. We can understand that. Yes, we can understand each other, and we can say, “This is a natural thing that happens when you get old.” If it were my daughter, she would tell me that the TV was too loud (when I turned up the volume). We can still comfort each other and say that it is okay with friends (in the ADC). For example, hearing aids can be used. “You have to get a something model. People actually using them tell me about them. I am grateful. My daughter does not know anything. (Daughter would say) “Mom, it is time for you to visit an otolaryngology (because) the TV is so noisy.” But here, I can take good care (from others like me).”

(ID: ID01)

“I, you know, in my hubris, in my assumptions, I thought ID04s going to die and not be taken care of in such a place… It was really arrogant thinking, (after receiving care here and observing other people being supported), now that I think about it. It is not that I did not think that as we get older, some parts of us will always get worse, and that we will need the help of others when that happens, but I somehow thought that I would be fine. That is why I have come to realize that the idea of life on pins and needles is an illusion.”

(ID: ID03)

(3) Have Trouble Knowing What to Do

Only ID04 said that she had trouble knowing what to do. She talked about “I have nothing to do” and “I have trouble because I have no idea what to do.”

“There are several types of (programs). But right now, I... do not want to do anything. I am not interested in anything. It is like I have been shut up and have forgotten how to go out…. I wondered where I would sit. In kindergarten, everyone has a chair at home, right? So, even if I sit down, I do not know where I should sit. There is a kind of confusion... Hmmm. First, when you enter school, you already know where you are supposed to sit, if it is your desk. In school, you already know where to sit at a desk. But in a place like this, from the chair to the desk, you do not know where to come and take your place. Unlike at school, I do not have a firm place to belong to.”

(ID: ID04)

(4) Put Oneself in a Shaded Exchange Relationship

All four participants reported their experiences of human relationships within the ADC. ID01, ID02, and ID03 showed their pleasure in “establishing friendships with certain people” but ID04 explained that she was now challenged to explore her place and pleasure within ADC.

“(When I am at home, all I do is watch TV, but not when I come to the ADC) That is good, is it not (ID01 waves to his friend, gesturing for him to talk)? Yes, talking about eating is the best; it is harmless. ...I do not really make eye contact with people I do not like. There may be some people, but I do not have any trouble because I only stay near people I can talk to.”

(ID: ID01)

“I do not know many people here. (I do not know many people. When I first came to ADC), I did not know where I was supposed to sit. So, I felt confused. But everyone was so kind that when I was just standing there and wondering where I should sit, they (staff and other clients) would tell me, “Hi XX (name of ID04), come over here and sit here.” … I am trying to figure out how to make myself fun like other people who can laugh and smile. That is what I am looking for.”

(ID: ID04)

ID02 and other people said that they held “small talk only.’’ Subsequently, she explained her chatting as “trivial.” She also enjoyed this experience. Researchers often observed laughing people who talked about non-serious, casual, and easy things. They enjoyed conversations about meals, weather, and their outfits.

“We do not talk about anything difficult, just trivial (laughs and smiles). (Researcher: Is it fun?) Yes, it is. … I think it is not good if I force myself to talk to the other person (deeply) about his/her condition when he or she is not feeling well with his/her health condition.”

(ID: ID02)

“(Relationships with old friends are already shut out. (Researcher: So, is this ADC where you can get your new friends, right?) Right. With my new friends, it is just superficial talk. I think it is very necessary to talk to people instead of spending a lonely day alone.”

(ID: ID03)

(5) Examine the value of elderly people in need of care in society

ID01 and ID03 explained their “worry about the burden on society of a system that supports those in need of care” owing to receiving care within ADC. ID03 was also thinking, to “explore what the elderly who need care can contribute to ADC.”

“It is a life of just being taken care of without being of any use to others; is not it? Instead, we can do something useful for each other. ... (ADC staff) does not only take care of the clients, but also makes them do something helpful. This would be nice for both of us.”

(ID: ID03)

(6) Savor My Regular Contact with Others

All participants explained or observed their feelings about something good in their regular contact with others.

ID02 smiled when talking about her routine plan to see a friend. We integrated the related codes into “making a promise to see others again.” Researchers observed that two other people (ID01 and ID03) also promised to meet in the next ADC visit with their friends and smiling.

“People who visit ADC are in (physically) bad condition. There were no healthy individuals here. This is why we do not meet each other externally. But for now, I am looking forward to meeting those people on any given day (smiling).”

(ID: ID02)

ID02 explained that she should be careful about self-care in weekly flamework. After the interview conversation, she additionally explained that routine ADC visits make her keep milestones in her daily life; therefore, we considered this as “getting milestones in one’s life framework.” In Japanese, she said the ADC visit was “MEYASU” and the authors translated it to mean “milestone.”

“When going out (for the ADC), one has to know the day, week, and time. If you lose track of it, you are in trouble. I always do everything by myself, and I think it is important.”

(ID: ID02)

ID01 and ID03 explained that “ADC is a good opportunity to go out on a regular basis.”

“It’s our regular outing, so come dressed nicely”, she said, grabbing the hem of my cardigan and welcoming me with a smile. (ID: ID01)

## 4. Discussion

It was found that the expectation of ADC to function as a social interaction tool was higher than the period before the COVID-19 pandemic. The context of ADC users was that it was difficult for them to communicate with others on a daily basis because of restrictions on going out and the difficulty of going out and using ICT. In a society with activity restrictions, ADC is a rare contact point for users.

Through interaction with others, participants were exposed to diverse feelings, which gave meaning to their lives. As a base of interaction with others, the experience of “put oneself in a shaded exchange relationship” and “Savor my regular contact with others” were merged. Prior studies have shown that access to the world through ADC activities improves clients’ psychosocial well-being [14], and social participation helps older adults feel more stimulated, confident, and content [15]. Similar to these results, ADC can be considered a place for sustaining accessibility to a social circle out of their own home. Under the restriction of going out and limited contact with others, knitting friendships with others and regular opportunities to contact others were the significant functions of ADC. Additionally, the development of friendships within limited and closed human groups in ADC may be good for clients’ quality of life. It was mentioned that prior to COVID-19, participants had enjoyed friendships not only in the neighborhood but also in ADC. The fact that this was restricted and that they could only have friendships at the ADC may have meant that their options were more limited. Socioemotional selectivity theory suggests that aged people tend to narrow the range of social partners, allowing people to conserve physical and cognitive resources and freeing time and energy for selected social relationships [16]. COVID-19 restricts their choice, and ADC cannot provide the opportunity to have contact with older adults who do not require nursing care. Japanese literature reviewed current discussions on ICT and human relationships, indicating that a superficial human relationship that avoids the risk of injuring or being hurt the opponent is required now, and a kind of “safe” relationship has advantages and disadvantages, and social networking system using ICT has minimized its disadvantages.” [17] However, the participants expressed difficulty in using current ICT. If this restricted situation continues, new and advanced technologies or service development may be required to maintain ADC clients’ social relationships. 

Within the ADC, participants “take a catastrophic defensive posture in situations where one’s perception of value is shaken” and they “express oneself positively to justify one’s daily life.” “Examine the value of elderly people in need of care in society” was a new experience no literature mentioned before. Participants sometimes expressed “we” instead of “I” in talking about their experiences within the ADC. They recognized themselves as a person with a disability, and they shifted their viewpoint from a disabled individual to a general group of “disabled older people.” The Japanese long-term care insurance system is supported by insurance fees and nation/municipality taxes [18]. They recognized themselves as individuals and sometimes as a part of a group of disabled old people, and they were threatened by the feeling of being a social burden. 

During the interviews and observations, the word “usefulness” emerged frequently. It seems so important to them that they were useful to others right now, and when faced with situations that threatened this, they became defensive, trying to gain a superior position through social comparison or by expressing their disadvantage clearly. Social comparison was observed among the UK nursing home residents; residents without dementia distanced themselves from residents with dementia by engaging in social comparison, and they regularly pointed out those with dementia and emphasized their own perceived cognitive superiority while also expressing sympathy and frustration over the repetitive or disruptive behaviors associated with severe dementia [19]. Usually, in a community care setting, clients experience “feeling simultaneously grateful and ashamed of oneself as a recipient of care services.” This context might emphasize the motivation to behave defensively within the ADC and justify their daily lives as care recipients. Our results also showed their attitude of “self-pity” within ADC. Self-pity is an emotional response that we experience from time to time [20], and a study of Japanese people showed that it can occur on a daily basis, not necessarily in serious or critical situations [18]. It includes not only pity for the self but also ambivalent attitudes of “distrust of pity” and “desire for compassion” toward others [21]. 

Since clients face the threat of identifying themselves as being “useless”, ADC should help prevent subsequent negative experiences. Notably, two participants expressed their pleasure in having the opportunity to express their self-pity in the interview. It could have a positive effect on nurses’ listening. A review paper on nurses’ listening attitudes in Japan suggested that nurses might build a relationship of mutual psychological stability by listening, accepting, and empathizing with patients’ past or present events [22]. Future research should explore how to provide better care for clients who experienced the feeling of being useless to prevent a negative impact on their health. Only one participant showed trouble with nothing to do/required within the ADC. She was the shortest-term client of ADC, and we could not identify the problem as a result of her personality/disability or shorter use history. It is possible that clients cannot express what they hope and how they face troubles. 

Only one participant, ID04, showed her trouble with having no idea what to do/hope. We cannot establish whether this experience was a result of her personality/disability or a shorter term of use. However, we should focus on the possibility that some clients would have trouble figuring out how to stay in the ADC. Due to their context of feeling ashamed of their own life and being recipients of care services, it might be difficult to freely enjoy their time within ADC. Japanese people were reported to act in accordance with norms that were considered appropriate for the situation at the time and keep in mind the interaction between themselves and other people in the situation [23]. Future research is required to determine how clients can find their schedules/hopes within the ADC.

The most prominent limitation of this study is the paucity of data. The number of participants was limited to four users attending one office in Tokyo, all of whom were female. Some were more severely disabled, and some were male; their experiences were not mentioned. It was also difficult to ask the participants directly for peer checking because of the new Omicron strains and the difficulty in conducting interviews using the online tool. There is a need to further refine this case study by increasing its number and introducing more user-friendly online communication tools. Despite these limitations, this study represents a new challenge in clarifying how elderly people in need of care in socially restrictive environments spend their time in ADC, a rare opportunity for social interaction, and how it can affect their daily lives.

## 5. Conclusions

With the spread of COVID-19, ADC clients faced a threat of a lack of social interaction opportunities. There were six categories pertaining to experience within ADC that would encourage ADC clients to defend themselves from the negative impact of weakened social ties. Scheduled regular ADC use was an opportunity to savor human contact for clients. To develop policy and practical responses to clients’ social restrictions during COVID-19, it is important to ensure the use of ADC as a safe place to interact with others, considering the novel needs created by the pandemic.

## Figures and Tables

**Table 1:** Summary of categories of clients’ background.

Category	Sub-Category
Restricted social interaction outside of ADC	COVID-19 restrictions on going out
	ADC is good place to stay
	Dropping out of the old familiar
	Difficulty in communicating using ICT
	Difficulties in interacting with others due to disability
	Difficulty in communicating with family and neighbors
Feeling simultaneously grateful and ashamed of oneself as a recipient of care services	Grateful to live with access to care services
	Feeling patronized for living with access to care services
	My future is going to be much worse

**Table 2:** Summary of categories of clients’ experiences with ADC.

Category	Sub-Category
Take a catastrophic defensive posture in situations where one’s perception of value is shaken	The painful experience of being confronted with the fact that I was disabled through the care I was receiving at the ADC
	Cynicism against the disabilities of others for self-protection
	Producing self-compassion actively
Express oneself positively to justify one’s daily life	Happy to express and be recognized that I am still useful and valuable
	Motivation to behave positively
	Getting a seed to have a conversation with family members
	Receiving care in the same place as other elderly people who need care, making it easier to accept one’s disabilities
Have trouble knowing what to do	I have nothing to do
	I have trouble because I have no idea what to do
Put oneself in a shaded exchange relationship	Establishing friendships with certain people
	Small talk only
Examine the value of elderly people in need of care in society	Worry about the burden on society of a system that supports those in need of care
	Explore what the elderly who need care can contribute to ADC
Savor my regular contact with others	Making a promise to see others again
	Getting milestones in one’s life framework
	ADC is a good opportunity to go out on a regular basis

## Data Availability

The data presented in this study are available on request from the corresponding author. The data are not publicly available due to privacy and ethical consideration.
